# HIF-1α induction during reperfusion avoids maladaptive repair after renal ischemia/reperfusion involving miR127-3p

**DOI:** 10.1038/srep41099

**Published:** 2017-01-20

**Authors:** Elisa Conde, Sara Giménez-Moyano, Laura Martín-Gómez, Macarena Rodríguez, M. Edurne Ramos, Elia Aguado-Fraile, Ignacio Blanco-Sanchez, Ana Saiz, María Laura García-Bermejo

**Affiliations:** 1 Biomarkers and Therapeutic Targets Lab, Hospital Universitario Ramón y Cajal, Instituto Ramón y Cajal de Investigación Sanitaria (IRYCIS), Madrid, Spain; 2 Pathology Department, Hospital Universitario Ramón y Cajal, Madrid, Spain

## Abstract

Ischemia/reperfusion (I/R) leads to Acute Kidney Injury. HIF-1α is a key factor during organ response to I/R. We previously demonstrated that HIF-1α is induced during renal reperfusion, after ischemia. Here we investigate the role of HIF-1α and the HIF-1α dependent mechanisms in renal repair after ischemia. By interference of HIF-1α in a rat model of renal I/R, we observed loss of expression and mis-localization of e-cadherin and induction of α-SMA, MMP-13, TGFβ, and collagen I. Moreover, we demonstrate that HIF-1α inhibition promotes renal cell infiltrates by inducing IL-1β, TNF-α, MCP-1 and VCAM-1, through NFkB activity. In addition, HIF-1α inhibition induced proximal tubule cells proliferation but it did not induce compensatory apoptosis, both *in vivo. In vitro*, HIF-1α knockdown in HK2 cells subjected to hypoxia/reoxygenation (H/R) promote cell entry into S phase, correlating with *in vivo* data. HIF-1α interference leads to downregulation of miR-127-3p and induction of its target gene Bcl6 *in vivo*. Moreover, modulation of miR-127-3p in HK2 cells subjected to H/R results in EMT regulation: miR127-3p inhibition promote loss of e-cadherin and induction of α-SMA and collagen I. In conclusion, HIF-1α induction during reperfusion is a protector mechanism implicated in a normal renal tissue repair after I/R.

Renal Ischemia/reperfusion (I/R) is at the basis of Acute Tubular Necrosis (ATN), as morphological expression of kidney injury after blood supply alterations. Acute Renal Failure as well as renal transplantation are two relevant clinical situations associated to ATN development after a renal I/R episode. In spite of the increasing knowledge of pathophysiological mechanisms triggered by I/R, efficient therapeutic interventions for ATN prevention, reduction or repair are not still available in clinical practice. Therefore, studies devoted to identify factors with potential therapeutical value are needed.

Hypoxia Inducible Factor (HIF) is a heterodimeric transcription factor and key regulator of cell responses to low oxygen conditions. Under low oxygen conditions, HIF accumulates and binds to the Hypoxia Response Elements (HRE) located in the promoter or enhancer region of target genes^1^,^2^. Although this is the canonical HIF-1α regulation, recent evidences demonstrate that HIF-1α could accumulate under several stimuli even in normoxic conditions as we have previously demonstrated^3^.

Regarding the kidney, HIF-1α is mainly expressed in tubular epithelial cells^4^ and it is the main controller of I/R response in proximal tubule cells. Indeed, ischemic preconditioning confers kidney protection against subsequent acute ischemic injury by activating HIF-1α and HIF-2α, among others. Moreover, accumulation of HIF-1α and HIF-2α by pharmacological inhibition of prolilhydroxylases (PHDs) administration to mice results in renal protection against ischemia. On the other hand, HIF-1α and HIF-2α heterozygous knockdown (KO) mice are more susceptible to I/R injury^5^,^6^. In a model of renal transplant in rats, donor pre-treatment with PHD inhibitor prevented graft injury and increased receptor survival^7^. Additionally, sustained HIF-1α expression has been involved in development of chronic kidney disease^8^.

There are hundreds of known HIF target genes. HIF-1α could promote cell proliferation^9^ but it can also inhibit cell proliferation by regulating directly cell cycle progression^10^,^11^ or it can also promote cell death through different mechanisms^12^. Previous work of our lab demonstrated that HIF-1α also controls miRNAs expression in renal I/R, particularly miR-127-3p *in vitro* and *in vivo*^13^. Moreover miR127 is a relevant mediator of proximal tubule cell response to ischemic damage by promoting cell adhesion^13^. miR-127-3p is also involved in the control of cell proliferation and cell death through apoptosis related targets including Bcl-6^14^.

Our previous studies demonstrated that HIF-1α is essential for proximal tubule cell survival during ischemia but also in reperfusion. HIF-1α inhibition by siRNA administration *in vivo* aggravates renal I/R injury and exacerbates proximal tubule damage^3^. Based on these findings, in this work we studied several mechanisms triggered by HIF-1α contributing to renal tissue repair after ischemic injury *in vivo*. We demonstrate that HIF-1α inhibition during reperfusion has a deleterious effect on renal injury recovery since this factor regulates the EMT process, the renal inflammation and the balance between proliferation and apoptosis, all contributing to an accurate renal tissue repair after I/R. NFkB activation, miR127-3p inhibition and Bcl6 increase upon HIF-1α inhibition, among other mechanisms, contribute to the abnormal renal tissue repair described here.

## Results

Previous data of our lab demonstrated that HIF-1α is stabilized in proximal tubule cells during ischemia and in late reperfusion. Moreover *in vivo* interference of HIF-1α leads to an exacerbated tissue damage and renal dysfunction^3^. In order to study the role and the mechanisms triggered by HIF-1α in I/R injury resolution, we efficiently interfered the HIF-1α protein and reduced HIF-1α activity in our model of renal I/R in the present work (Supplementary Figures S1 and S2).

**Figure 1:**
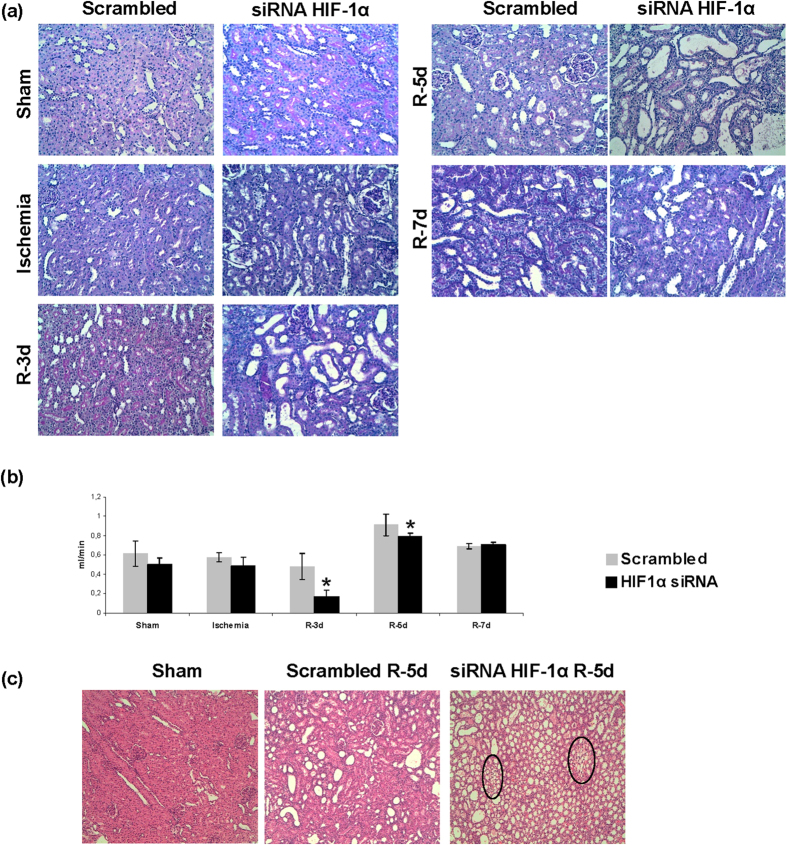
HIF-1α inhibition during reperfusion aggravates ischemic renal damage and leads to peritubular capillary proliferation. (**a**) PAS staining of paraffin-embedded renal tissue from I/R rats where HIF-1α was interfered or scrambled control (n = 5 in each experimental group). Renal structure is affected during I/R mainly at the proximal tubule fraction by loss of microvilli and tubule dilatation. HIF-1α interfered rats exhibited aggravation of renal damage. Images magnification: 200X. (**b**) Creatinine clearance as renal function estimation is shown. HIF-1α interfered rats exhibited lower creatinine clearance as indication of worsening in renal function. Asterisks indicate statistical significance (*P < 0.05, +/− s.d.). (**c**) H&E staining of paraffin-embedded renal tissue sections allow the observation of vascular networks. As observed, HIF-1α interference promotes the proliferation of peritubular capillary. Images magnification: 100X. Experimental chirurgic model, embedded paraffin tissue blocks and storage, PAS and H&E staining and renal function estimation were performed during 2011.

HIF-1α interference during reperfusion increased tubular damage at 3–5 days (Fig. 1a, Table 1). Moreover renal function is worsened 3 days after ischemia measured by creatinine clearance (Fig. 1b). Additionally we find that HIF-1α inhibition promotes peritubular capillary proliferation at 5 days of reperfusion as the appearance of vascular networks in renal cortex suggests (Fig. 1c).

### HIF-1α interference promotes EMT and fibrosis markers expression

To study the role of HIF-1α in the renal tissue repair after ischemia we use our *in vivo* model of HIF-1α interference in renal I/R in rats.

**Figure 2:**
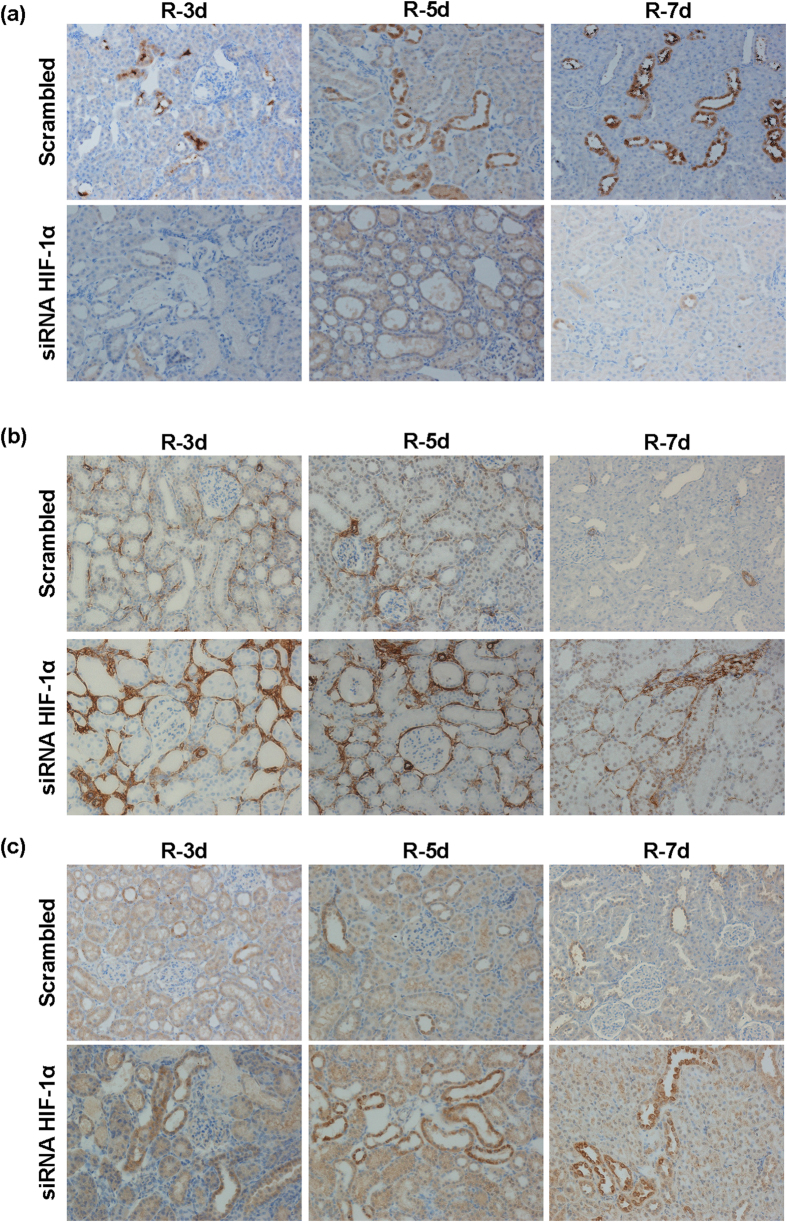
HIF-1α interference promotes EMT markers expression. Expression and localization of the EMT markers e-cadherin, α-SMA and MMP13 was determined by immunohistochemistry, in paraffin-embedded renal tissue sections from I/R rats where HIF-1α was interfered or scrambled control (n = 5 in each experimental group). Images magnification: 200X. (**a**) e-cadherin: HIF-1α interfered rats exhibited lower expression of e-cadherin compared to scrambled group and e-cadherin is re-distributed from intercellular localization in proximal tubule cells. (**b**) α-SMA: HIF-1α interfered rats exhibited a higher expression of α-SMA, mainly localized at renal parenchyma, compared to scrambled group. (**c**) MMP13: HIF-1α interfered rats exhibited a higher expression of MMP13, mainly localized at proximal tubule cells, compare to scrambled group. Experimental chirurgic model and embedded paraffin tissue blocks and storage, after chirurgical procedure, were performed during 2011. Tissue sections and IHC of e-cadherin, α-SMA and MMP13 were performed during 2016.

**Figure 3:**
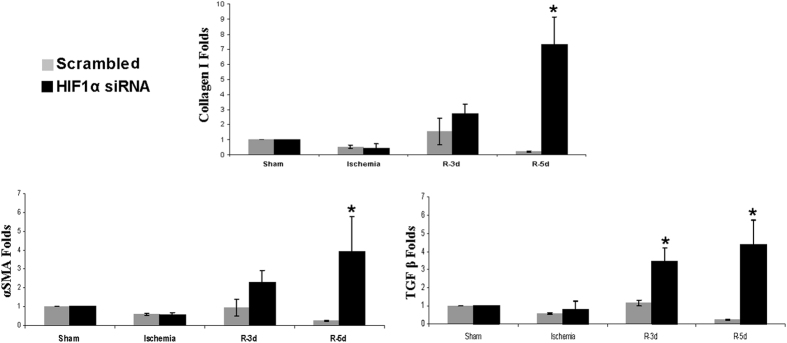
HIF-1α inhibition promotes pro-fibrotic factors expression. Expression of the pro-fibrotic factors TGF-β, αSMA and collagen I was estimated by qRT-PCR in renal tissue from I/R rats (n = 5 in each experimental group). Data are expressed as fold change using 28S levels as reference. Asterisks indicate statistical significance (*P < 0.05, +/− s.d.). As it can be observed, HIF-1α interference leads to upregulation of these pro-fibrotic factors. Experimental chirurgic model was performed during 2011, RNA extraction and qRT-PCR were performed during 2011–2012.

Abnormal repair of the kidney epithelium by induction of EMT among others processes has been described as contributing to fibrosis development and chronic damage as a long-term outcome of a maladaptive renal tissue repair. For this reason, we studied the expression of EMT markers including e-cadherin, α-SMA and MMP13, by immunohistochemistry and fibrosis mediators such as TGF-β, α-SMA and collagen I, by qRT-PCR in our interfered rats. The results shown in Fig. 2 demonstrate that e-cadherin expression is reduced in proximal tubules from interfered rats and redistribution from intercellular localization is also observed (Fig. 2a). In addition, induction of α-SMA expression (Fig. 2b) and MMP13 (Fig. 2c) is detected in these interfered rats. Semi-quantitative estimations of EMT markers immunostaining are shown in Table 2. In agreement, qRT-PCR in total renal lysates demonstrated an increase expression of collagen I, α-SMA and TGF-β, in HIF-1α interfered rats compared with Scrambled, at 5 days after ischemia (Fig. 3).

All these findings together demonstrated that HIF-1α absence in renal tissue during reperfusion after ischemia promotes an EMT process and triggers an initial fibrosis, contributing to an abnormal repair of I/R injury.

### HIF-1α interference exacerbates inflammatory response after I/R

**Figure 4:**
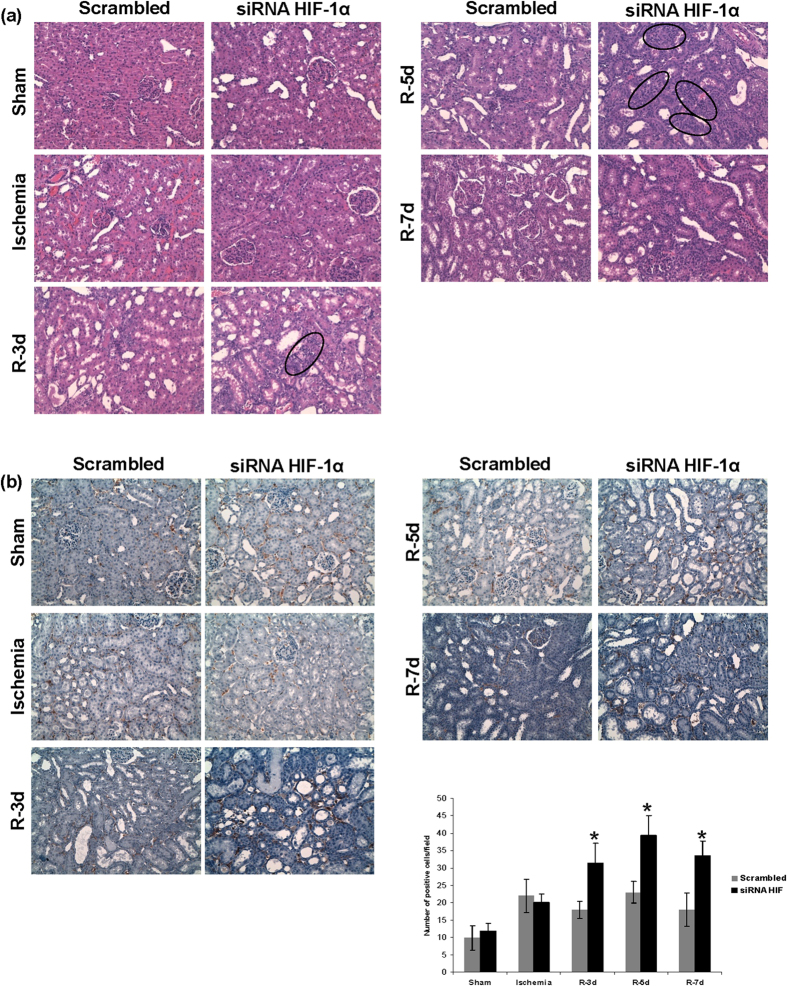
HIF-1α inhibition during reperfusion stimulates inflammatory response. (**a**) H&E staining of paraffin-embedded renal tissue sections from I/R rats where HIF-1α was interfered or scrambled condition (n = 5 in each experimental group). These images evidence cell infiltrates are more prominent in HIF-1α interfered rats (marked by circles) than in scrambled condition. Images magnification: 300X (**b**) T cell marker immunohistochemistry and quantification of paraffin-embedded sections from I/R rats. As showed, T cell amount is higher in HIF-1α interfered rats at 3-5-7 days of reperfusion. Asterisks indicate statistical significance (*P < 0.05, +/− s.d.) Images magnification: 300X. Experimental chirurgic model, embedded paraffin tissue blocks, block storage and H&E staining were performed during 2011. Tissue sections and IHC of T-cell marker were performed during 2011–2012.

**Figure 5:**
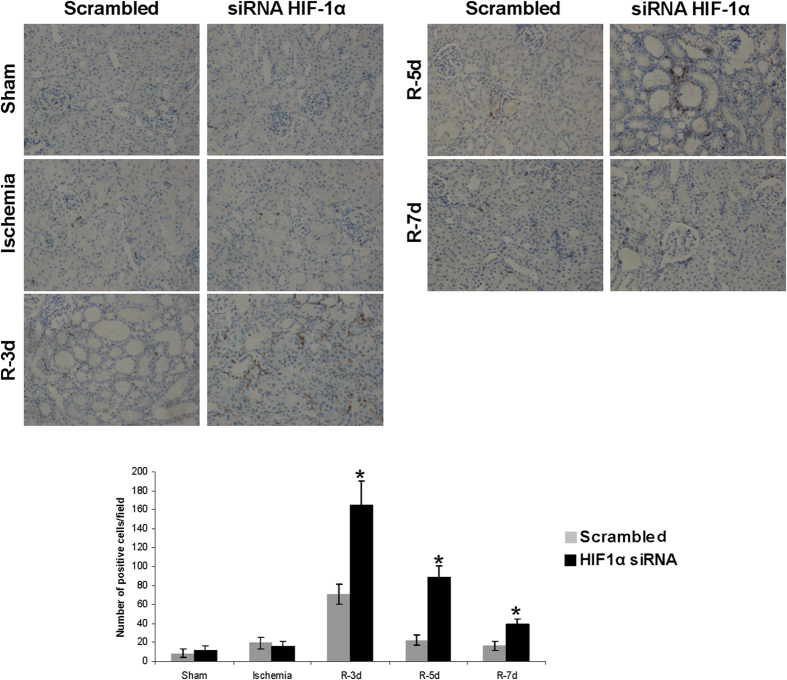
HIF-1α interference promotes macrophages infiltration after I/R. CD68 immunohistochemistry in paraffin-embedded sections from I/R rats and quantification of positive cells per field is shown. As it can be observed, the amount of CD68 positive cells is higher in HIF-1α interfered rats, at 3-5-7 days of reperfusion. Asterisks indicate statistical significance (*P < 0.05, +/− s.d.) Images magnification: 300X. Experimental chirurgic model, embedded paraffin tissue blocks and storage were performed during 2011. Tissue sections and IHC of CD68 were performed during 2011–2012.

**Figure 6:**
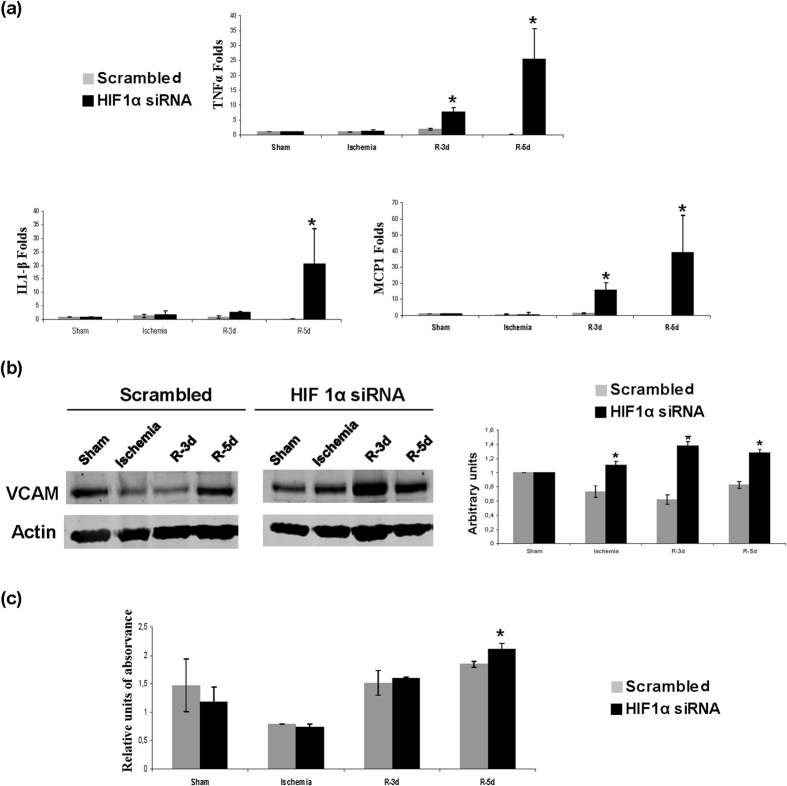
HIF-1α inhibition during reperfusion promotes pro-inflammatory mediators. (**a**) Expression levels of the pro-inflammatory mediators IL-1β, MCP-1 and TNFα in renal tissue estimated by qRT-PCR. Data are expressed as fold change using 28S levels as reference. As it can be observed, IL-1β, MCP-1 and TNFα expression is upregulated in HIF-1α interfered rats (n = 5 in each experimental group). Asterisks indicate statistical significance (*P < 0.05, +/− s.d.) (**b**) VCAM-1 protein levels estimated by western blot. Actin protein levels were used as protein loading control. Cropped blots are displayed. Quantification of three independent western blot is also shown. Asterisks indicate statistical significance (*P < 0.05, +/− s.d.). As observed VCAM-1 protein expression is higher in HIF-1α interfered rats. (**c**) NFκB P65 activity in kidney tissue nuclear extracts of HIF-1α interfered rats estimated by ELISA. These results show an increase of NFκB P65 activity at 5 days of reperfusion in HIF-1α interfered rats compare to scrambled group. Asterisks indicate statistical significance (*P < 0.05, +/− s.d.). Experimental chirurgic model was performed during 2011. RNA extraction, qRT-PCR, protein extraction and western blot were performed during 2011–2012. For NFκB P65 activity, protein extraction and ELISA assay were performed in 2011.

Next, since the inflammatory response underlies I/R, we estimated and characterized the infiltrates in renal tissue. Haematoxylin-eosin staining (Fig. 4a), T Cell maker (Fig. 4b) and CD68 macrophage marker (Fig. 5) immunohistochemistry in renal tissue demonstrate an increase of inflammatory infiltrates during reperfusion at 3, 5 and 7 days in HIF-1α interfered rats compared to scrambled. In correlation with this, we analysed pro-inflammatory gene expression including TNFα, IL-1β and MCP-1, in renal tissue lysates by qRT- PCR. These genes were markedly induced 5 days after ischemia in HIF-1α interfered rats compared to scrambled (Fig. 6a). In addition, we have studied the endothelium activation through VCAM-1 expression. We observed an induction of VCAM-1 3 days after ischemia in HIF-1α interfered rats compared with scrambled Quantification of VCAM-1 protein expression in three independent experiments is also shown. (Fig. 6b and Supplementary Figure S3).

Rationally, we have determined NF-κB activity, as one of the major mediators of inflammation, and we have observed an increase of NF-κB activity at 5 days post ischemia in HIF-1α interfered rats, correlating with the pro-inflammatory genes induction described above (Fig. 6c).

Taken together these results demonstrated that HIF-1α absence exacerbates the inflammatory response in renal tissue during reperfusion through NF-κB activation, among other mechanisms, maybe contributing to an anomalous renal tissue repair.

### HIF-1α inhibition promotes unbalanced proximal tubule cell proliferation

**Figure 7:**
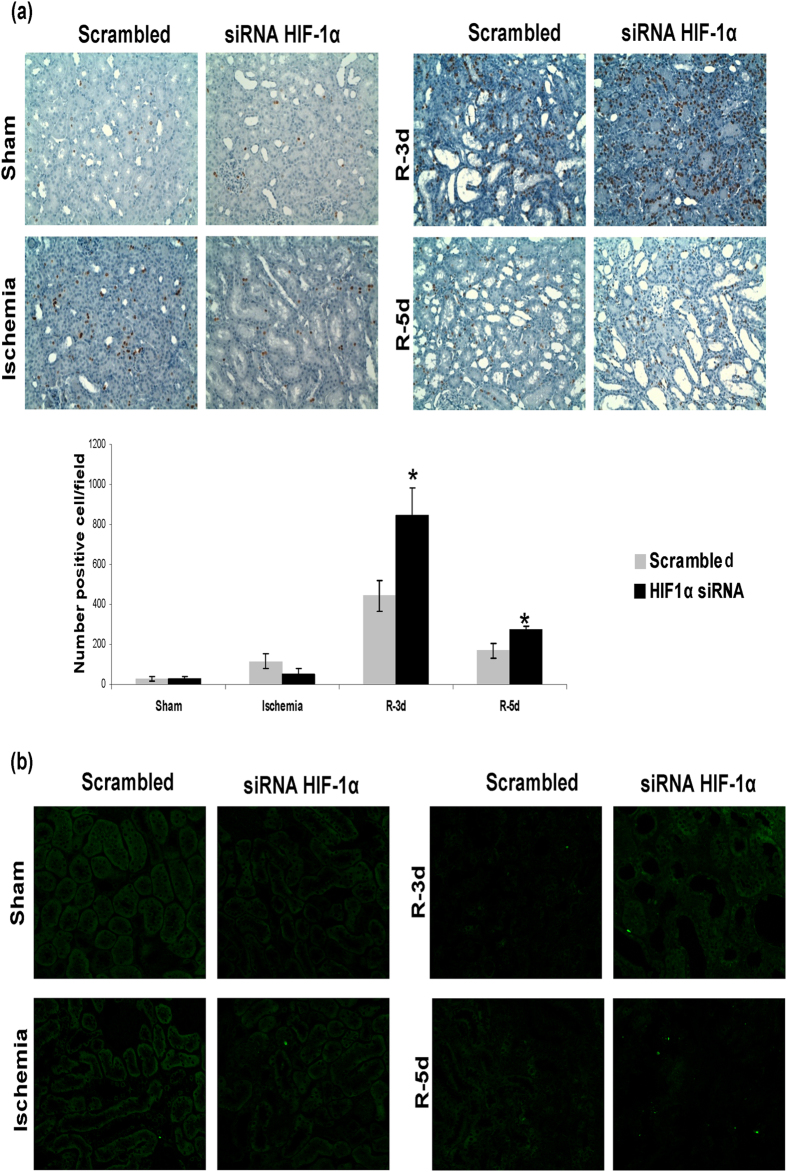
HIF-1α inhibition exacerbates proximal tubule cell proliferation and unbalances proliferation vs apoptosis. (**a**) Cell proliferation in I/R rats was estimated by BRdU injection. BRdU incorporation in renal cells was estimated by immunohistochemistry for BRdU in paraffin-embedded renal tissue and later quantification. As observed, proliferation of proximal tubule cells was enhanced in HIF-1α interfered rats (n = 5 in each experimental group). Asterisks indicate statistical significance (*P < 0.05, +/− s.d.). Images magnification: 400X (**b**) Cell apoptosis was estimated by TUNEL staining in paraffin-embedded renal tissue. As observed, there is not difference in apoptotic cell number between both experimental groups. Images magnification: 200X. Experimental chirurgic model and embedded paraffin tissue blocks and storage were performed during 2011. Tissue sections for BrdU detection as well as IHC were performed during 2011–2012. Tissue sections for TUNEL and the assay were performed during 2012.

A correct balance between proliferation of surviving cells and apoptosis is one of the most important mechanisms involved in a correct repair of ischemic damage. Therefore we investigated the role of HIF-1α in proximal tubule cells proliferation in our model of renal I/R. By means of BrdU incorporation and immunohistochemistry in renal tissue, we observed exacerbated cell proliferation, 3–5 days after ischemia, in HIF-1α interfered rats compare with scrambled. (Fig. 7a).

In addition, we studied proximal tubule cell apoptosis by means of the TUNEL assay in renal tissue. We did not observe proximal tubule cells apoptosis in HIF-1α interfered rats neither in scrambled group at 3 and 5 days of reperfusion (Fig. 7b).

**Figure 8:**
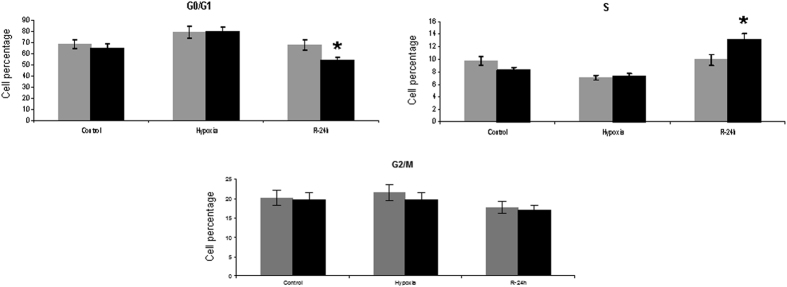
HIF-1α knockdown during reperfusion in proximal tubule cells HK2 promotes cell cycle progression. HIF-1α was interfered by specific siRNA in HK-2 cells and 48 h later, cells were submitted to H/R protocol. Distribution of HK2 cells in the cell cycle phases was determined by means of propidium iodide staining and flow cytometer analysis (n = 3 in each experimental group). Quantification of cell percentage in each cell cycle phase was performed by Modfit 2.0 software (Beckton Dickinson). The percentage of cells in S replicative phase is significantly increased in HIF-1α knockdown cells compare to scrambled group, at 24 h of reoxygenation. Asterisks indicate statistical significance (*P < 0.05, +/− s.d.). Cell culture, *in vitro* HIF-1α inhibition and cell cycle distribution assay were performed during 2011.

Trying to unveil the relationship between HIF-1α and the proximal tubule proliferation observed here, we have interfered this transcription factor, by transfection of specific siRNA, in the human proximal epithelial tubule cells (HK2) subjected to the hypoxia/reoxygenation, a protocol which reproduced *in vitro* the stimuli and effects of renal I/R *in vivo*^15^. HIF-1α was efficiently abrogated in HK-2 cells (95% of protein reduction in comparison with scrambled transfected cells) and we determined the cell cycle phase distribution by propidium iodide incorporation and flow cytometry. The results shown in Fig. 8 demonstrate that HIF-1α knockdown promotes proliferation of HK2 cells at 24 h of reoxygenation, since cells from G0/G1 progress to S replicative phase, as the significant changes in cell percentages indicate.

All these data demonstrate that HIF-1α inhibition lead to loss of balance between proximal tubule cells proliferation and apoptosis, by promoting cell cycle progression and consequently contributing to an abnormal repair.

### HIF-1α inhibition affects miR-127-3p expression during I/R repair

microRNAs have emerged as crucial and tight regulators of the cellular response to insults. Previous data from our lab described miR-127-3p as key mediator of proximal epithelial tubule cell response to I/R^13^. Thus, we studied the role of HIF-1α in the regulation of miR-127-3p as a possible mechanism contributing to the repair of renal I/R injury.

**Figure 9:**
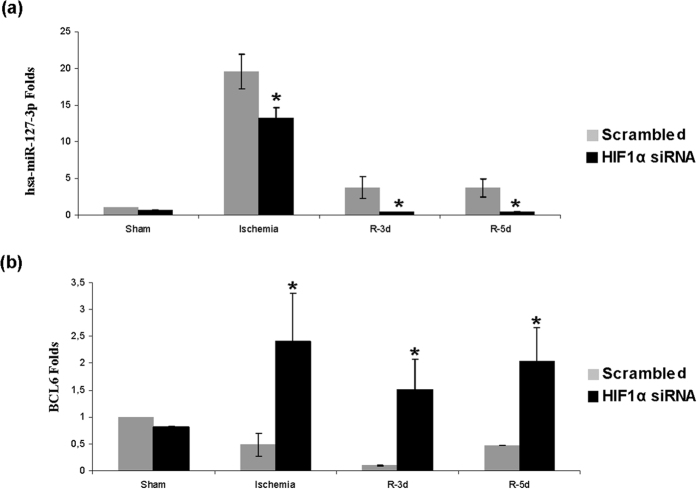
HIF-1α inhibition lead to miR-127-3p expression decrease and Bcl6 expression enhance. Expression of miR-127-3p and its target gene Bcl6 was estimated by qRT-PCR in renal tissue from I/R rats (n = 5 in each experimental group). Data are expressed as fold change using RNU6B and 5S mean and 28S levels as references in miR-127-3p and Bcl6 respectively. Asterisks indicate statistical significance (*P < 0.05, +/− s.d.). HIF-1α interference leads to miR-127-3p expression decrease during ischemia and at 3–5 days of reperfusion as compared to scrambled control. Consequently, we found an upregulation of miR-127-3p target gene Bcl6 at the same times. Experimental chirurgic model, RNA extraction and qRT-PCR were performed during 2011.

By means of qRT-PCR we analysed the expression of miR-127-3p and its validated target gene Bcl6. These results showed a decrease of miR-127-3p levels during ischemia and at 3–5 days of reperfusion in HIF-1α interfered rats compared with Scrambled (Fig. 9a). Accordingly, Bcl6 expression increased at the same time (Fig. 9b), indicating that miR-127-3p and Bcl6 are possible mediators of HIF-1α in the renal repair in our model.

**Figure 10:**
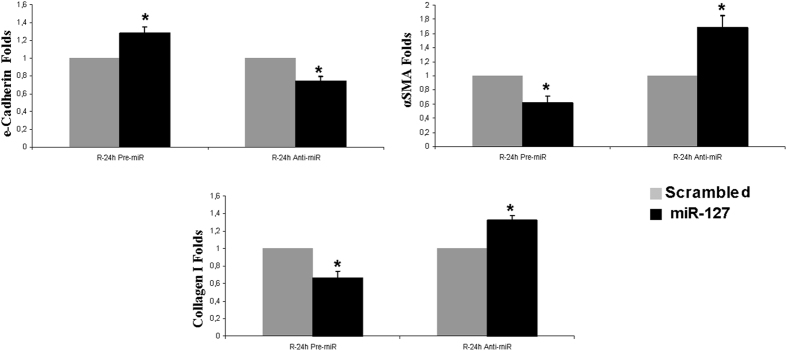
miR-127-3p inhibition in HK2 cells promotes EMT markers expression. miR-127-3p was modulated by transfection of specific pre-miR127-3p and anti-miR127-3p and 48 h later, cells were subjected to the H/R protocol. Expression of the EMT markers e-cadherin, α-SMA and collagen I in HK2 cells was estimated by qRTR-PCR in cell lysates (n = 3 in each experimental group). Data are expressed as fold change using 28S levels as reference. Asterisks indicate statistical significance (*P < 0.05, +/− s.d.). miR-127-3p inhibition leads to a loss of e-cadherin and to an increase of α-SMA and collagen I expression, at 24 h of reoxygenation, in comparison to scrambled group. The over expresion of miR-127-3p exhibited the opposite results: increase in e-cadherin and reduction in α-SMA and collagen I expression. Cell culture, *in vitro* miR-127-3p modulation, RNA extraction and qRT-PCR were performed during 2016.

Moreover, trying to investigate the involvement of miR127-3p in the abnormal repair exhibited by HIF-1α interfered rats, we modulated miR127-3p using specific Pre-miR127-3p and anti-miR-127-3p in human proximal tubule cells HK2 and we estimated the expression of EMT markers including e-cadherin, α-SMA and collagen I, by qRT-PCR. The miRNA modulation was efficient: pre-miR127-3p/pre-scrambled, 140 ± 15 folds; anti-miR-127-3p/anti-scrambled: 5 ± 0,5 folds. The results shown in Fig. 10 indicate that inhibition of miR127-3p leads to a reduction in e-cadherin mRNA expression and to an increase of α-SMA and collagen I expression. The overexpression of miR127-3p reverses these effects.

All these findings demonstrate that miR-127-3p is a HIF-1α effector during the renal tissue repair after ischemia, among others.

## Discussion

We have previously reported that HIF-1α is induced at ischemia and during reperfusion in a rat model of renal I/R^3^. In the present work we demonstrate that HIF-1α is induced during reperfusion in order to assure precise renal tissue regeneration after ischemic damage. Indeed HIF-1α inhibition: (i) promotes the EMT process and increased expression of fibrotic markers, might be priming chronic damage; (ii) exacerbates inflammatory response through NFk-β activation and pro-inflammatory factors induction, leading to increasing inflammatory infiltrates; (iii) unbalances proximal tubule cells proliferation vs apoptosis. Some of these alterations are mediated by miR-127-3p which is downregulated when HIF-1α is knocked down. All these features are associated with an abnormal renal repair, proving that HIF-1α induction during reperfusion is essential for an accurate ischemic damage restoration. In fact, damaged kidney can regenerate its normal architecture after ischemic injury in many of the cases but frequently cellular responses to the insult become maladaptive and lead to long term renal impairment^16^.

Oxygen imbalance leading to renal tissue hypoxia occurs in patients with Acute Kidney Injury (AKI) and with chronic kidney disease (CKD). Moreover several meta-analyses have demonstrated that AKI episodes could lead to CKD later on^17^. Additionally, several studies in CKD animal models suggest that HIF-1α is suppressed by factors including oxidative stress and uremia and underlies the pathogenesis of CKD^18^. HIF-1α has been extensively described as a key factor for cell adaptation to hypoxia, including renal tissue^19^. Consistently with our previous results^3^ and the findings presented here, the activation of HIF-1α post-ischemia by pharmacological treatment with PHDs inhibitors significantly reduces renal I/R injury^20^. Pre-ischemic targeting of the PHD/HIF pathway also demonstrated beneficial effect in prevention of chronic renal damage, including fibrosis, subsequent to acute damage^6^.

Related to fibrosis, our immunohistochemistry results demonstrate that HIF-1α inhibition during reperfusion promote the epithelial mesenchymal transition process since we clearly observed the loss of e-cadherin expression and mis-localization in proximal tubules, the increase of α-SMA expression in renal parenchyma, most probably related to proliferation and activation of resident fibroblasts and proximal tubule cells transitioning, and the increased MMP13 expression in proximal tubules, confirming the acquisition of mesenchymal and migratory phenotype by transitioning proximal tubule cells. Moreover our gene expression results confirm the initiation of fibrosis since induction of TGF-β, as a critical fibrotic mediator, and induction of Collagen I, as an anomalous renal extracellular matrix component, are both observed. It has been widely demonstrated that maintained HIF-1α activation aggravates chronic renal hypoxic injury by promoting pro-fibrotic factors as well as EMT in a rat models^21^ and epithelial cell cultures^22^. Moreover, correlation of HIF-1α accumulation and fibrotic areas in chronic kidney damage without hypoxic environments has been also observed^23^. In our system, the abrogation of HIF-1α expression could promote proximal epithelial cell dysfunction and EMT process contributing to renal fibrosis. Remarkably, it has been recently reported that HIF-1α and HIF-2α ko mice exhibited enhanced I/R associated fibrosis and infiltration^6^. The absence of the HIF-1α dependent genes would enhance the maladaptive renal regeneration after ischemia allowing a chronic renal damage as a long-term outcome of renal I/R episode.

It has been also proposed that mechanisms triggered or regulated by HIF-1α enhance tissue repair during recovery from ischemic injury including Erythropoietin (EPO)^24^ or stromal cell-derived factor-1 (SDF-1)^25^, that promote recruitment of progenitor cells to regenerating tissues. However, proliferation and differentiation of proximal tubule cells non-lethally damaged is the major contributor to ischemic injury regeneration^26^. Our results in rats demonstrate that HIF-1α interference during reperfusion exacerbates epithelial tubule cell proliferation although apoptosis to compensate this proliferation is absent. Moreover, *in vitro* experiments in proximal tubules cells HK2 confirmed that the knockdown of HIF-1α directly affected cell proliferation since higher proportions of HK-2 cells went into S-Phase from G1, assuring cell division. It has recently reported that HIF-1α induction during hypoxia inhibits mesenchymal stem cells proliferation by increasing the cell cycle inhibitor p27^27^. It could be conceivable that the absence of HIF-1α in our models leads to modulation of cell cycle regulators, including p27 inhibitor, and cell proliferation could take place.

On the other hand, it is well known that appropriated ischemic damage repair during acute kidney injury resolution involves an accurate balance of proximal tubule cells proliferation vs apoptosis, involving classical signaling pathways^28^. Our results indicate that in HIF-interfered rats, after an exacerbated proximal tubule cell proliferation, compensatory apoptosis does not occur, contributing to an abnormal renal tissue repair. HIF-1α has been described as pro-apoptotic and anti-apoptotic regulator through Bcl-2 family genes, targeting mitochondria enzymes or by means of p53 interactions^29^. Although we did not study mechanisms regulating apoptosis in this work, we could not discard that any HIF-dependent pathway related to cell death and affected by HIF-1α abrogation could also contribute to the apoptosis abolition described here.

Recently, miRNAs have been involved in the cell response to I/R in kidney^30^. Related to this, here we demonstrated that *in vivo* HIF-1α interference leads to a lower expression of miR-127-3p and, consequently, increased expression of its target gene Bcl6, a transcriptional repressor involved in apoptosis^31^ and survival regulation^32^. Previous results of our lab demonstrated that miR127-3p is regulated by HIF-1α during proximal epithelial cell response to ischemia and it is a protector mechanism against renal ischemic injury^13^. Since HIF-1α interfered rats, where miR127-3p is reduced, exhibited EMT induction and initial fibrosis, miR127-3p also appears here as a protector mediator against renal chronic damage establishment. Accordingly with our results, other groups demonstrate that miR-127-3p inhibits cell proliferation^14^,^33^, migration and invasion^34^. Indeed our *in vitro* results demonstrate that miR127-3p regulates the expression of EMT and fibrotic markers. To our knowledge none of these markers are validated targets of miR-127-3p even though MMP13 is a predicted target for this miRNA. However, interestingly it has recently published that BCL6 can regulate EMT reducing e-cadherin by enhancing the expression of its transcriptional repressor ZEB1^35^. Therefore, miR-127-3p could regulate EMT and fibrosis directly, affecting not yet validated targets, or indirectly through Bcl6, among other mechanisms.

On the other hand, our results demonstrate that HIF-1α interference promotes the appearance of extensive infiltrates in the renal parenchyma, composed of T cells and macrophages as the immunohistochemistry for T cell marker and CD68 confirmed. These infiltrates are consistent with the expression of the pro-inflammatory mediators MCP-1, IL-1β and TNFα and with endothelium activation. According to the induction of pro-inflammatory genes, we also show that HIF-1α inhibition promotes NFkB activation during reperfusion. Several reports indicate a crosstalk between HIF-1α and NFkB^36^. Related to our results, it has been recently reported that miR-127-3p suppressed NFkB activity in HCC cells, also promoting HCC proliferation and tumor-associated inflammation^37^. MCP-1, IL-1β and TNFα have been extensively linked to renal infiltration after injury including ischemic injury and tubular cells appears as the main source of these chemokines^38^. In our animal model, HIF-1α interference enhance MCP-1, IL-1β and TNFα levels, accordingly with data recently published demonstrating that a conditional knockout mouse for HIF-1α exhibited enhance renal inflammatory response in I/R and UUO *in vivo* models, by activation of leucocytes and endothelial cells^6^. The MCP-1, IL-1β and TNFα induction observed in our work are most probably due to the NFkB activation also detected. However other mechanisms involved in these inductions can not be discarded. Thus, molecular relationship between HIF-1α and IL-1β has been reported in glioblastoma cells, where IL-1β decreased HIF-1α levels and promoted apoptosis^39^. Moreover it has been published that HIF-1α can induce TNF α in a model of osteoclast under hypoxia^40^. In addition, VCAM-1 has been also described as a NFkB modulated gene^41^. Up to this moment, there are no reports validating MCP-1, IL-1b, TNF-α or VCAM-1 as miR-127-3p targets genes. However, miR127 appears as an important miRNA for inflammatory regulation response, in particular for macrophages response^42^. In fact, miR127 regulates IL-6 and TNF-α cytokine release by macrophages in lung inflammation since the IgG FcγRI (CD64) is a target of miR-127-3p^43^. Moreover miR127-3p can also regulate macrophages polarization in lung^44^. Macrophages control the long-term outcome of acute ischemic damage^45^ exhibiting phenotype shift during the kidney repair processes^46^. We cannot discard that the absence of miR127-3p in HIF-1α interfered rats could also contribute to the abnormal renal tissue repair by modulating macrophages responses and phenotypes shift.

In summary, our work demonstrates that HIF-1α induction during reperfusion is critical for an adequated renal repair process after ischemic injury. HIF-1α abrogation during reperfusion leads to a maladaptive restoration of the proximal tubule epithelia and the renal parenchyma. Our work unveils HIF-1α dependent mechanisms, including miR127-3p, responsible for adaptive processes of repair since their inhibition results in abnormal restoration. These HIF-1α dependent mechanisms and processes include EMT and pro-fibrogenic factors induction, unbalanced proximal epithelial cells proliferation vs apoptosis and promotion of inflammation process. Deregulation of all these processes by HIF-1α abrogation could contribute to AKI long term outcome alterations including the development of progressive fibrotic kidney disease. The mechanisms described here could allow designing novel and targeted therapeutic strategies, including HIF-1α and miR127-3p based strategies, in order to promote adaptive renal recovery and to minimize chronic kidney alterations after acute injury, as an outstanding aim in clinical Nephrology^47^.

## Methods

### I/R model in rat. BrdU treatment

Male Sprague-Dawley rats (180–200 g) from our own colony were divided in groups of 5–7 animals for each condition. Animals were treated according to the Spanish guidelines (RD 53/2013) that are in compliance with the EU Guide for the Care and Use of Laboratory Animals (UE63/2010). The experimental protocol was approved by the Internal Comitee for Animal Ethics of Hospital Universitario Ramón y Cajal.

Rats were anesthetized with an inhaled anaesthesia mixture of 2% isoflurane (Abbott Laboratories Ltd., Queenborough, Kent, England) and 1 l/min oxygen and placed on a temperature-regulated table (37 °C). Renal ischemia was performed by clamping both renal pedicles during 45 min. Sham-operated group underwent the same surgical procedure without clamping. Animals were sacrificed at different times of reperfusion and kidneys were harvested.

For proliferation studies, 250 mg/Kg of BrdU (Sigma-Aldrich) were injected intraperitoneally 24 hours before animal sacrifice.

These animals were generated and also used in our previous published work^3^.

### Cell culture, H/R *in vitro* protocol

HK-2 cells (ATCC) were cultured in DMEM/F12 containing 10% FBS, 1 g/l insulin, 0.55 g/l transferrin, 0.67 mg/l selenium (Invitrogen), 2 mM glutamine, 100 U/ml penicillin and 100 μg/ml streptomycin (Invitrogen), in a humidified atmosphere with 5% CO2 at 37 °C. Cells were cultured until confluence and then they were serum deprived for 24 hours. Monolayers were cultured for 6 hours in HBSS (Invitrogen), in a hypoxic atmosphere containing 1% O_2_, 94% N_2_, 5% CO_2_ (Air Liquide). After hypoxia, cells were maintained in complete medium and 21% O_2_ for reoxygenation^15^. Serum-starved cells following 6 h in HBSS were used as Control.

### HIF-1α siRNA transfection *in vitro* and *in vivo*

HK2 at 70% of confluence were transfected with 100 nM of 4 different HIF-1 α siRNAs (Customized Silencer Select siRNAs: S225214, S225215, S225216 Ambion; siRNA sc-44225, Santa Cruz Biotechnologies) and scrambledd siRNA (sc-37007, Santa Cruz Biotechnologies), using Lipofectamine 2000 according to the manufacturer’s protocol. siRNA sc-44225 (Sta. Cruz) was selected for the *in vitro* experiments due to its high efficiency. Transfected cells were subjected to H/R after 48 h of transfection.

*In vivo*, siRNA sc-44225 containing 3 sequences against 3 different HIF-1α exons was used for HIF-1 α inhibition. 75 μg/kg body weight of siRNA mixed with Jetpei (Polyplus, Genycell) following manufacturer´s instructions was injected to animals, through the tail vein, before ischemia in sham and ischemia groups or during reperfusion in R-3d, R-5d and R-7d groups, as previously described^3^.

### Pre-miRs and Anti-miRs Transfection *in vitro*

HK2 cells at 70% confluence were transfected with 100 nM anti-miR-127 (MM10400, Anti-miR 127-3p miRNA Inhibitor, Life Technologies, Madrid, Spain) y anti-miR-Scrambled (miRVana miR inhibitor Negative ControlAntimiR #1, Life Technologies). Pre-miR-127 (miRVana miRNA mimic miR-127-3p MC10400, Life Technologies) and Pre-miR-Scrambled (miRVana miRNA mimic Negative Control #1, Life Technologies) were transfected with a final concentration of 0.1 nM, using Lipofectamine 2000 according to the manufacturer’s protocol. Transfected cells were subjected to H/R after 48 h of transfection.

### Periodic acid–Schiff (PAS) and Hematoxilin-eosin staining, immunohistochemistry and TUNEL assay

Renal tissue was fixed in 4% phosphate-buffered formalin (pH 7.4), and 3μm sections of paraffin-embedded kidneys were stained with PAS, H&E or immunostained with anti-BrdU (Dako, Barcelona, Spain), Tcell marker (Santa Cruz sc-52711), anti-CD-68 (Santa Cruz sc-59103), anti-e-cadherin (Santa Cruz sc-7870), anti-alpha SMA (Abcam ab7817), anti-MMP13 (Abcam ab9012) or anti-HIF-1α (Santa Cruz sc-10790) as described previously^3^. Semi-quantitative evaluation of staining was performed by pathologists in a double-blind study, including estimation of staining extension and staining intensity (Surface/intensity). TUNEL assay was performed following the manufacturer’s instructions (DeadEnd™ Fluorometric TUNEL System, PROMEGA).

### Cell cycle phases distribution assay

For each sample, attached cells were trypsinized. Cell suspensions were stained with 50 μg/ml propidium iodide (PI) in PBS containing 0.1% NP40.

Cell cycle distribution was determined using a Beckton Dickinson FACScan flow cytometer, analysing 20.000 cells per sample. The percentage of cells in G_0_-G_1_ phase, S phase or G_2_-M phase were estimated using Modfit 2.0 software (Beckton Dickinson).

### Quantitative RT-PCR and primers

Renal tissue or HK2 cells were lysed into 1 ml of Tri-Reagent (Ambion). Total RNA was extracted and quantified using Nanodrop 2000 (Thermo Scientific). cDNA was obtained from 2 μg of total RNA from each sample using Transcriptor First Strand Synthesis kit (Roche) and 1 μl of cDNA sample was used as template for PCR with LightCycler 480 Sybr Green I Master (Roche) following the manufacturer’s instructions. PCR were carried out in Lightcycler 480 equipment (Roche). For each sample and experiment, triplicates were run and normalized by 28S mRNA levels. Primer pairs used are shown in Table 3.

miRNAs were detected and quantified by Real-Time PCR using Universal RT miRNA PCR System (Exiqon). 200 ng of the RNA was used as template for retrotranscription in a final volume of 20 μl. cDNA was diluted 1/11 with nuclease-free sterile water and 4 μl was used as template for PCR reaction.

Real time PCR detection was performed using SYBR Green (Roche) and specific commercially available probes (Exiqon). Master Mix preparation and temperature cycles were performed following manufacturer’s instructions. The mean of RNU6B and ribosomal RNA 5 s expression was used as internal control for data normalization.

All reactions were carried out by triplicates in Light Cycler 480 equipment (Roche) and miRNA expression were calculated using 2^nd^derivative method (2^−ΔΔ^*C*_T_) (Light Cycler 480 Software 1.5, Roche) and expressed as fold change.

### Western blotting

Western blotting in tissue was performed as previously described^48^. Antibodies working dilutions were: 1/200 for VCAM1 (Santa Cruz Biotechnology) and 1/2000 for total actin (Sigma).

### HIF-1α and NFκB P65 transcription factor activity assays

HIF-1α and NFκB P65 activity studies in nuclear extracts from rat kidney tissues, were performed using HIF-1α and NFκB P65 transcription factor assay kit, based on binding to specific adsorbed-oligo’s and ELISA detection method and following manufacturer’s instructions (HIF-1α: 47096; NFκB P65: 40096. Active motif, Transam).

### Statistical analysis

Data are presented as mean ± SD. After the Levene test of homogeneity of variance, the Kruskal-Wallis test was used for group comparison. A p < 0.05 was considered significant. In case of significant differences, intergroup differences were analysed by post-hoc Mann-Whitney U tests with the Bonferroni correction. Statistical analysis was carried out using Statistical Package for the Social Sciences (SPSS) version 15.0.

## Additional Information

**How to cite this article:** Conde, E. *et al*. HIF-1a induction during reperfusion avoids maladaptive repair after renal ischemia/reperfusion involving miR127-3p. *Sci. Rep.* **7**, 41099; doi: 10.1038/srep41099 (2017).

**Publisher's note:** Springer Nature remains neutral with regard to jurisdictional claims in published maps and institutional affiliations.

## Supplementary Material

Supplementary Information

## Figures and Tables

**Table 1:** Histopathological damage evaluation in HIF-1α interfered rats.

Histopathological damage		
	Scrambled	HIF-1α siRNA
**Sham**	−	−
**Ischemia**	+	++
**R-3d**	+	++++
**R-5d**	++	+++
**R-7d**	+	+

Histopathological damage was evaluated as double-blind study based on the following characteristics: (1) Morphological changes in proximal tubular epithelium: necrotic cells, brush border loss, cell detachment; (2) Infiltrate presence; (3) Hyaline cylinders presence. (4) Other alterations also considered were: tubular dilatation, interstitial edema and medular congestion.

**Table 2:** Immunohistochemistry semi-quantitative evaluation.

	E-Cadherin		aSMA		MMP13	
	Scrambled	HIF1α siRNA	Scrambled	HIF1α siRNA	Scrambled	HIF1α siRNA
**R-3d**	+/++	0/0	++/++	+++/+++	+/+	++/+
**R-5d**	++/++	0/0	+/++	+++/+++	+/+	++/+++
**R-7d**	++/+++	+/+	0/0	++/++	+/+	++/+++

Semi-quantitative evaluation by pathologists was performed as double-blind study based on surface and intensity staining. (Surface/intensity).

**Table 3:** Primers sequences used in qRT-PCR for genes.

GENE	PRIMER	SECUENCE 5′–3′
**28S**	Forward	CAGTACGAATACAGACCG
	Reverse	GGCAACAACACATCATCAG
**HIF**-**1 α**	Forward	TCCTATGTGCTGGCTTTGG
	Reverse	AGTGTACCCTAACTAGCCG
**TGF**-**β**	Forward	CAACAATTCCTGGCGTTACC
	Reverse	TATTCCGTCTCCTTGGTTCAG
**α**-**SMA**	Forward	ACCCAGATTATGTTTGAGACC
	Reverse	AGAGTCCAGCACAATACCAG
**IL**-**1β**	Forward	CACCTCTCAAGCAGAGCACAG
	Reverse	GGGTTCCATGGTGAAGTCAAC
**TNFα**	Forward	CCAGGAGAAAGTCAGCCTCCT
	Reverse	TCATACCAGGGCTTGAGCTCA
**MCP**-**1**	Forward	CTGCTACTCATTCACTGGC
	Reverse	CTTCTGGACCCATTCCTTATTG
**Collagen**	Forward	GTGGAAACCTGATGTATGCT
	Reverse	TGGTGATACATATTCTTCTGGG
**BCL6**	Forward	CCGCTACAAAGGCAACCTC
	Reverse	AGCGGTAGGGTTTTTCACCT
**E**-**Cadherin** (**human**)	Forward	ACACCAACGATAATCCTCCGA
	Reverse	CATCAGCATCAGTCACTTTCAG
**α**-**SMA** (**human**)	Forward	GAGAAGAGTTACGAGTTGCC
	Reverse	ATGATGCTGTTGTAGGTGGT
**Collagen I** (**human**)	Forward	CTGGAAAGAATGGAGATGATGG
	Reverse	CCAAACCACTGAAACCTCTG
