# Supplementary material for: HIF-1α induction during reperfusion avoids maladaptive repair after renal ischemia/reperfusion involving miR127-3p
Source: Sci Rep. 2017 Jan 20;7:41099. doi: 10.1038/srep41099 (PMC5247697; doi:10.1038/srep41099)
Supplement: Supplementary Information [file srep41099-s1.pdf]

Supplementary Information

Title: HIF-1α induction during reperfusion avoids maladaptive repair after renal ischemia/reperfusion involving miR127-3p

Authors: Elisa Conde[1], Sara Gimenez-Moyano[1], Laura Martín-Gomez[1], Macarena Rodríguez[1], Edurne Ramos[1], Elia Aguado-Fraile[1], Ignacio Blanco-Sanchez[1], Ana Saiz[2] and María Laura García Bermejo [1,3]

Addresses: [1] Biomarkers and Therapeutic Targets Lab, Hospital Universitario Ramón y Cajal, Instituto Ramón y Cajal de Investigación Sanitaria (IRYCIS), Madrid, Spain.

[2]Pathology department, Hospital Universitario Ramón y Cajal, Madrid, Spain

[3] **Address correspondence to**: M. Laura García-Bermejo, PhD

Biomarkers and Therapeutic Targets Unit

Instituto Ramón y Cajal de Investigación Sanitaria (IRYCIS)

Crta. Colmenar, Km 9,100

28034, Madrid, Spain

Phone: +34-913368075

Fax: +34-913369016

Email: garciabermejo@gmail.com

Supplementary figure S1.

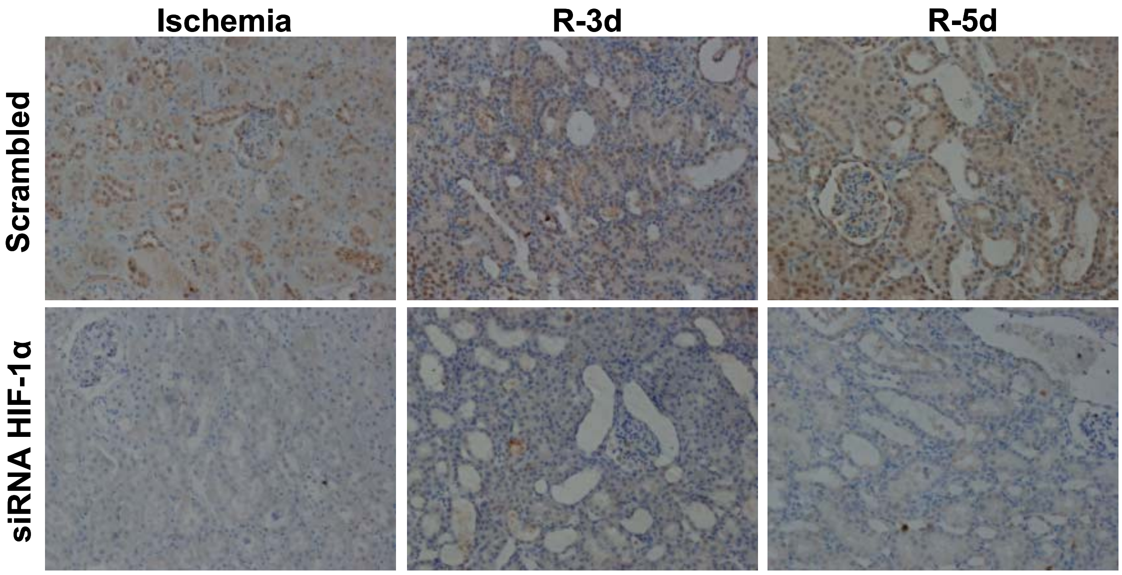

S1. HIF-1α interference *in vivo.* HIF-1α protein interference was estimated by immunohistochemistry in paraffin embedded renal tissue sections. Lower expression os HIF-1α in renal tissue is observed in HIF-1α interfered rats compare with scrambled. (n=5 in each experimental group). Representative images are shown. Magnification 300x. Experimental chirurgic model, embedded paraffin tissue blocks and storage, tissue section and IHC of HIF-1α were performed during 2011.

Supplementary figure S2.

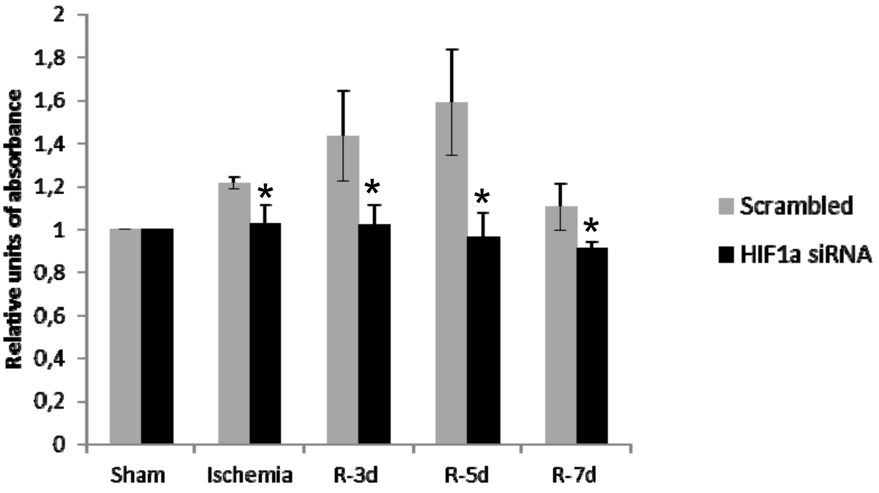

S2. **HIF-1α interference protein control.** HIF-1α protein expression was estimated by means of ELISA in I/R kidney lisates from HIF-1α interfered rats compare to scrambled (n=5 in each experimental group). Asterisks indicate statistical significance (*P<0.05, +/- s.d.). Experimental chirurgic model, protein extraction and HIF-1α ELISA were performed during 2011.

Supplementary figure S3.

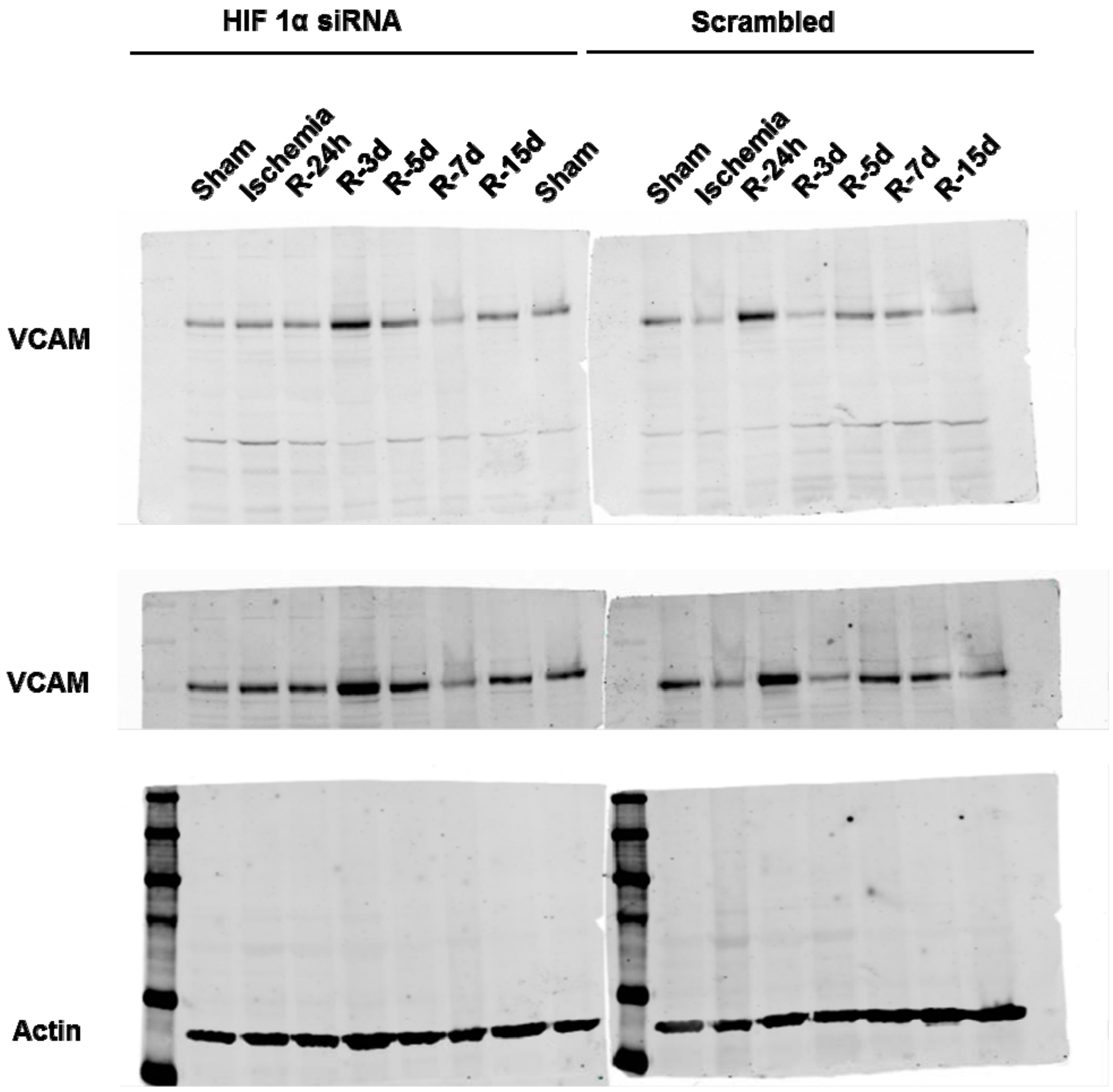

S3. **Full-length blot of VCAM1 and Actin expression.** VCAM1 and actin protein levels were estimated by western blot in I/R kidney lysates from HIF-1α interfered rats and scrambled rats. A longer exposure of VCAM1 blot is also shown. Experimental chirurgic model was performed during 2011. Protein extraction and western blot were performed during 2011-2012.
